# Assessment of reproducibility of a VP7 Blocking ELISA diagnostic test for African horse sickness

**DOI:** 10.1111/tbed.12968

**Published:** 2018-08-02

**Authors:** Manuel Durán‐Ferrer, Montserrat Agüero, Stephan Zientara, Cécile Beck, Sylvie Lecollinet, Corinne Sailleau, Shirley Smith, Christiaan Potgieter, Paloma Rueda, Patricia Sastre, Federica Monaco, Ruben Villalba, Cristina Tena‐Tomás, Carrie Batten, Lorraine Frost, John Flannery, Simon Gubbins, Baratang A. Lubisi, José Manuel Sánchez‐Vizcaíno, Michelle Emery, Tracy Sturgill, Eileen Ostlund, Javier Castillo‐Olivares

**Affiliations:** ^1^ Laboratorio Central de Veterinaria LCV Madrid Spain; ^2^ UMR Laboratoire de Santé Animale ANSES INRA ENVA Maisons‐Alfort France; ^3^ Deltamune Pretoria South Africa; ^4^ Department of Biochemistry Centre for Human Metabolomics North‐West University Potchefstroom South Africa; ^5^ INGENASA Madrid Spain; ^6^ IZS dell'Abruzzo e Molise Teramo Italy; ^7^ The Pirbright Institute Pirbright UK; ^8^ OVI Pretoria Gauteng South Africa; ^9^ Universidad Complutense of Madrid Madrid Spain; ^10^ USDA Ames Iowa; ^11^ Present address: Department of Veterinary Medicine University of Cambridge Madingley Road Cambridge CB3 0ES UK

**Keywords:** African horse sickness, antibodies, diagnostic sensitivity, diagnostic specificity, ELISA, performance characteristics, reproducibility, ring trial

## Abstract

The laboratory diagnosis of African horse sickness (AHS) is important for: (a) demonstrating freedom from infection in a population, animals or products for trade (b) assessing the efficiency of eradication policies; (c) laboratory confirmation of clinical diagnosis; (d) estimating the prevalence of AHS infection; and (e) assessing postvaccination immune status of individual animals or populations. Although serological techniques play a secondary role in the confirmation of clinical cases, their use is very important for all the other purposes due to their high throughput, ease of use and good cost‐benefit ratio. The main objective of this study was to support the validation of AHS VP7 Blocking ELISA up to the Stage 3 of the World Animal Health Organization (OIE) assay validation pathway. To achieve this, a collaborative ring trial, which included all OIE Reference Laboratories and other AHS‐specialist diagnostic centres, was conducted in order to assess the diagnostic performance characteristics of the VP7 Blocking ELISA. In this trial, a panel of sera of different epidemiological origin and infection status was used. Through this comprehensive evaluation we can conclude that the VP7 Blocking ELISA satisfies the OIE requirements of reproducibility. The VP7 Blocking ELISA, in its commercial version is ready to enter Stage 4 of the validation pathway (Programme Implementation). Specifically, this will require testing the diagnostic performance of the assay using contemporary serum samples collected during control campaigns in endemic countries.

## 1 INTRODUCTION

African horse sickness (AHS) is an infectious but noncontagious viral disease affecting all species of *Equidae* and is caused by an *Orbivirus* of the family *Reoviridae* and characterized by respiratory and circulatory syndromes. Nine different serotypes of AHS virus (AHSV) have been identified. AHS is transmitted by species of *Culicoides* spp biting midges. All serotypes of AHS occur in eastern and southern Africa, from where they occasionally spread into countries surrounding the Mediterranean and on occasions reaching India and Pakistan (Mellor & Hamblin, 2004; Zientara, Weyer, & Lecollinet, 2015). Due to its high mortality rate, which can exceed 90% in susceptible populations, the huge economic losses that it causes in endemic countries and when it spills over into nonendemic territories, and its negative impact on international trade of equids, AHS is an OIE (World Organization for Animal Health)‐listed disease. Furthermore, its control is considered a priority and AHS is one of the six animal diseases currently included by the OIE in the procedure for official recognition of a country's disease‐free status (OIE, 2017a).

Clinical signs are characteristic but not pathognomonic of the disease, especially in endemic countries where the infection can cause a milder clinical form of the disease. Confirmatory diagnosis in the laboratory is most often achieved through virus detection techniques in blood samples or in postmortem tissue samples when AHSV‐infected horses die before specific antibodies can become detectable in clinical serum specimens (Mellor & Hamblin, 2004; Zientara et al., 2015). Currently, polymerase chain reaction (PCR) methods are the first choice for diagnosis (Agüero et al., 2008; Guthrie et al., 2013). Once the disease has been confirmed, virus characterization can be attempted by PCR typing techniques (Bachanek‐Bankowska et al., 2014; Weyer et al., 2015) or by the classical pathway of virus isolation in cell cultures followed by virus neutralization testing (OIE, 2017b).

Horses that survive natural infection develop antibodies against the infecting serotype of AHSV within 8–12 days postinfection and, consequently, serology is the most practical approach for surveillance in nonendemic countries, determining disease freedom in a population or for import–export testing prior to international trade. In endemic countries, where many horses are routinely vaccinated and/or experience reinfections, the use of serology for diagnosis, import/export or surveillance, requires the analysis of serum samples collected sequentially over a period of time to determine whether an increase or decrease in antibody titres are indicative of AHSV infections have occurred in the animals involved. Several laboratory procedures have been used for such purposes, including complement fixation (McIntosh, 1956; OIE, 2017b), agar gel immunodiffusion (Verwoerd, Huismans, & Erasmus, 1979), immunofluorescence or virus neutralization tests (Hamblin et al., 1991; Hopkins, Hazarati, & Ozawa, 1966; Mellor & Hamblin, 2004; OIE, 2017b). However, ELISA methods have prevailed and are currently considered the first choice because they are rapid, easy to standardize, easy to perform and have high‐throughput capacity (Hamblin, Graham, Anderson, & Crowther, 1990; Laviada, Roy, & Sánchez‐Vizcaíno, 1992; Maree & Paweska, 2005; OIE, 2017b; Rubio et al., 1998; Wade‐Evans, Wolhouse, O'Hara, & Hamblin, 1993).

The VP7 protein AHSV is the outer core protein of the capsid and a group‐specific antigen (Zientara et al., 2015; Maree & Paweska, 2005). The VP7 Blocking ELISA detects specific antibodies against the antigenically conserved VP7 protein. The test is currently commercially available INGEZIM AHSV COMPAC PLUS 2.0 (Ingenasa, Madrid, Spain). An indirect ELISA detecting VP7 protein is also available (Maree & Paweska, 2005). Both tests are recommended by OIE for serological diagnosis of AHS (OIE, 2017b) as well as for the implementation of checks prior to international movement of equids. Furthermore, the VP7 Blocking ELISA is one of the serological tests prescribed for the control of movement and importations to the European Union, according to requirements of Directive 2009/156/EC, as last amended (European Union, 2009).

To achieve harmonized criteria for validation of diagnostic tests, the OIE has proposed an assay validation pathway (OIE, 2017c) with the aim of declaring that a test is “fit for purpose”. This statement indicates the test is suitable and reliable for: (a) demonstrating freedom from infection in a population, animals or products for trade; (b) assessing the efficiency of eradication policies; (c) laboratory confirmation of clinical diagnosis; (d) estimating the prevalence of AHS infection; and (e) assessing postvaccination immune status of individual animals or populations. The validation pathway consists of four stages: Stage 1, determination of analytical characteristics (analytical sensitivity, specificity and repeatability estimates); Stage 2, determination of diagnostic characteristics (diagnostic sensitivity and specificity estimates); Stage 3, determination of reproducibility estimates; and Stage 4, declaration of fitness for purpose and international recognition (OIE, 2012).

The main objective of this study was to support the validation of the VP7 Blocking ELISA up to Stage 3 of the OIE validation pathway. For this, a “ring” trial, which included the participation of OIE Reference Laboratories and other specialist diagnostic laboratories, was set up to test a panel of field serum samples of different epidemiological origin and experimental sera from horses infected with AHSV and/or vaccinated with live attenuated, inactivated or recombinant AHSV vaccines. The inter‐laboratory reproducibility of the test was assessed by studying the Kappa agreement statistic (Thrusfield, 1995), *Z*‐score index (ISO, 2005) and variation of the test's performance (sensitivity and specificity) amongst the different laboratories. Through this comprehensive evaluation, we can conclude that the VP7 Blocking ELISA assay satisfied the OIE requirements of reproducibility and maintained adequate diagnostic performance characteristics.

## 2 MATERIALS AND METHODS

### 2.1 Participant laboratories

The study was organized by the Laboratorio Central de Veterinaria (LCV), Algete, Madrid, Spain (EURL/OIE Reference Laboratory for AHS) under the coordination of The Pirbright Institute, Pirbright, UK (OIE Reference Laboratory for AHS). Other participant institutes in the project were: (a) Agence nationale de sécurité sanitaire de l'alimentation, de l'environnement et du travail (ANSES), Maisons‐Alfort, France; (b) Onderstepoort Veterinary Institute (OVI) (OIE Reference Center for AHS), South Africa; (c) Deltamune, Centurion, South Africa; (d) VISAVET, University Complutense of Madrid, Spain (OIE Reference Center for AHS); (e) INGENASA, Madrid, Spain; (f) National Veterinary Services Laboratory (NVSL), USDA, Ames, Iowa, USA; and (g) Istituto Zooprofilattico Sperimentale dell'Abruzzo e Molise, Italy.

### 2.2 Diagnostic test kits

The test kits used in the study were obtained directly from the commercial manufacturer (INGENASA, Madrid, Spain). All partners used the same serum panel (Table 1) and the same tests. All the test kits belonged to the same manufacturing batches.

**Table 1:** Composition of panel of serum sample

Animal Species	Status of donor	Virus serotype	Time of collection	Expected value^a^	Number of samples
AHS noninfected					
Various	Not previously exposed to AHSV	Na	Na	Neg (*n* = 41)	Mule (11); Donkey (10); Horse (20)
Vaccinated and not infected with AHSV					
Horse	Vaccinated with attenuated vaccine	S‐4; S‐5	152‐428 dpv	Neg (*n* = 12); Pos (*n* = 42)	54
Horse	Vaccinated with inactivated vaccine	S‐1, S‐2, S‐4, S‐9	15‐115 dpv	Pos (*n* = 31)	31
Horse	Vaccinated with MVA‐VP2 vaccine	S‐9	42 dpv	Neg (*n* = 4)	4
Horse	Vaccinated with subunits vaccine	Na	52 dpv	Pos (*n* = 1)	1
Infected					
Horse	Naturally infected	Nd	Na	Pos (*n* = 19)	19
Horse	Experimentally infected	S‐2; S‐9	18‐28 dpi	Pos (*n* = 4)	4
Vaccinated and infected with AHSV					
Horse	Vaccinated with attenuated vaccine and experimentally infected	S‐4; S‐5; S‐6	152 dpv/67dpi	Pos (*n* = 11)	11
Horse	Vaccinated with inactivated vaccine and experimentally infected	S‐1, S‐2, S‐4, S‐9 (Vac)/S‐2 (Infec)	129‐143 dpv; 14‐28 dpi	Pos (*n* = 9)	9
Horse	Vaccinated with inactivated vaccine and experimentally infected	S‐9 (Vac)/S‐9 (infec)	116 dpv; 7‐14 dpi	Pos (*n* = 8)	8
Horse	Vaccinated with MVA‐VP2^a^ vaccine and experimentally infected	S‐9 (Vac)/S‐9 (Infec)	28 dpi	Pos (*n* = 4)	4
			Total	Neg (*n* = 57); Pos (*n* = 129)	186

Notes

dpi: days postinfection; dpv: days postvaccination; infec: infection; MVA: Modified Vaccinia Ankara viruses expressing single African horse sickness virus VP2 antigens; *n*: number of serum samples; na: nonapplicable; Na; not available; nd: no data; Nd: not done; Neg: negative; Pos: positive; Vac: vaccination.

a Expected value for the samples according to the original results obtained, by the VP7 Blocking ELISA, at the laboratory of origin.

The VP7 Blocking ELISA (INGEZIM AHSV COMPAC PLUS)*,* is designed to detect specific AHSV anti‐VP7 antibodies in sera from animals of any equine species, that is, horses, donkeys, zebra and their crosses. The principle of this test is the blocking of the reaction between the recombinant VP7 protein adsorbed to the ELISA plate and a peroxidase‐conjugated AHS‐VP7‐specific monoclonal antibody (Mab). VP7‐specific antibodies present in serum samples block the reaction between the antigen and the conjugated Mab resulting in a reduction in enzymatic reaction between the peroxidase and the colorimetric enzyme substrate added to the plate. The optical density readouts of the reactions were determined and results expressed as the percentage of reactivity between the conjugate and the antigen that is blocked by the serum sample.

The tests were conducted as indicated by the manufacturer's instructions. Briefly, serum samples and positive and negative controls were diluted in dilution buffer (PBS; 0.35 M NaCl, 0.05% Tween 20; 0.1% Kathon) and 100 μl/well added to two duplicate wells. After 1 hr incubation at 37°C the plates were washed five times with washing buffer (PBS; 0.135 M NaCl, 0.05% Tween 20). This was followed by addition of 100 μl/well of horseradish peroxidase‐labelled VP7‐specific Mab (supplied by manufacturer). After an incubation period of 30 min at 37°C, the plates were washed five times in washing buffer and 100 μl/well of the chromogenic substrate ABTS were added to all wells and incubated for 10 min after which the reaction was stopped by the addition of 100 μl/well of a 2% SDS solution. The optical density at 405 nm was read and results recorded. Mean values for each pair of samples were calculated, inclusive of positive and negative controls. The blocking percentage was calculated as follows: BP = [OD Neg Control − OD Sample] × 100/[OD Neg control − OD Pos Control]. Samples showing BP values >50% were considered positive; samples with BP <45% were considered negative; samples with values between 45% and 50% were doubtful.

### 2.3 Panel of serum samples

All participant laboratories contributed to the preparation of the sera panel by providing available samples. The panel was assembled at the LCV‐Algete, and it was made out of 186 serum samples representing all possible AHS sero‐epidemiological status that can be found in the field (Table 1): (a) AHS noninfected; (b) vaccinated (with either attenuated or inactivated whole virus vaccines, MVA‐VP2 vaccine, or recombinant subunit vaccines) and not infected with AHSV; (c) naturally or experimentally infected with AHSV; and (d) vaccinated (with either attenuated or inactivated whole virus vaccines or MVA‐VP2 vaccine) and infected with AHSV. Samples were sent by donor laboratories to LCV‐Algete and then collected, aliquoted and frozen at −20°C until they were distributed to each participant laboratory, blindly coded for testing. Once results were made available, they were collated and a database was created. Laboratory codes (codes A, B, C, D, E, F, G, H and I) were not broken until the exercise was completed.

### 2.4 Assay of samples, interpretation of results and expected values

In each laboratory, the VP7 Blocking ELISA test was conducted in duplicate for each serum sample. Samples were classified, according to the manufacturer's instructions, as positive, doubtful or negative. The criterion of maximum sensitivity was selected for the final test evaluation. Thus, doubtful results were considered as positive.

To determine the diagnostic characteristics of tests (sensitivity and specificity), a reference value was assigned to each sample tested. The reference value assigned was the result previously obtained by the VP7 Blocking ELISA in the laboratory that donated the serum.

### 2.5 Statistical methods

The assessment of reproducibility of the assays was the main objective of the study by estimating test's sensitivity (Se) and specificity (Sp) and their associated 95% confidence level interval (95% CI) calculated over the data generated by laboratories as a whole (total observations), after rejecting outlier laboratories.

For identifying outliers, two statistical approaches were complementarily used: (a) Cohen's Kappa statistic, used to compare the level of agreement of the results for each pair of laboratories (Thrusfield, 1995); and (b) The *Z*‐Score or standard score, which determines the level of standard deviation by which the value of an observation is below/above the mean value (ISO, 2005). Observed values above the mean have positive standard scores, while values below the mean have negative standard scores. *Z*‐score ranges from −3.0 standard deviations (which would fall to the far left of the normal distribution curve) up to +3.0 standard deviations (which would fall to the far right of the normal distribution curve). If a *Z*‐score is 0, it represents the score is identical to the mean score. When *Z*‐score values for a particular laboratory were above +2.0 or below −2.0, the laboratory's accuracy was considered questionable and its results were excluded from further analysis.

## 3 RESULTS

Tests were completed successfully by all participants and results were sent to LCV‐Algete who compiled the data and distributed it to all partners for sharing information and discussion.

**Figure 1:**
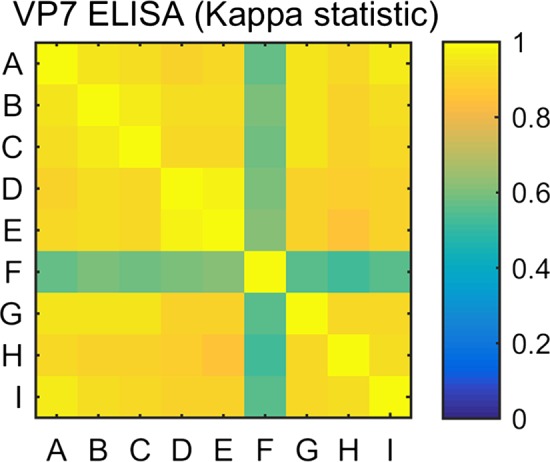
VP7 Blocking ELISA: inter‐laboratory reproducibility estimates by Cohen's Kappa factor statistic test [Colour figure can be viewed at wileyonlinelibrary.com]

The performance of laboratories was assessed by computing kappa statistics (Figure 1). This revealed that Laboratory F produced highly discordant results in comparison with the rest of the laboratories. Except for laboratory F, the results show that the VP7 Blocking ELISA had good or very good reproducibility between the participant laboratories (Kappa ≥ 0.7). The *Z*‐Scores computed from the data were consistent with the kappa statistics. Thus, the *Z*‐score range for laboratory F was −2.7 to 1.1, whereas the *Z*‐score range for the rest of laboratories was −1.4 to 1.1. For calculations of average sensitivity, data from laboratory F were excluded.

The sensitivity of the test in naturally or experimentally infected animals (not vaccinated) was 98.4% (181/184 [95% CI: 95.3–99.7]). If individuals were immunized prior to infection, sensitivity varied depending on the vaccine used. For animals previously vaccinated with vaccines containing VP7 antigen, the sensitivity was very high, both for individuals that received attenuated vaccines (Se: 100% [88/88; 95% CI: 95.9–100]) and for horses vaccinated with inactivated vaccines (Se: 100% [136/136; 95% CI: 97.3–100]). But when animals were not previously exposed to VP7 antigen (as with Modified Vaccinia Ankara [MVA] viruses expressing single AHSV VP2 antigens), detection of VP7 antibodies after infection was poor 34.4% (11/32 [95% CI: 18.6–53.2]). In vaccinated, noninfected animals, the sensitivity was 94.8% (561/592 [95% CI: 93.5–97.1]) with no major differences between types of vaccine used. The overall sensitivity (not vaccinated and infected plus vaccinated infected animals) of the test was 94.7% (977/1032 [95% CI: 93.1–96.0]).

The specificity of the VP7 Blocking ELISA was 100% (328/328; [95% CI: 98.9‐100]) in naïve animals (AHS noninfected animals), but lower in vaccinated and not infected individuals, reaching a value of 96.9% (124/128 [95% CI: 92.2‐99.1]). The overall specificity was very high 99.1% (452/456 [95% CI: 97.8‐99.8]; Table 2).

**Table 2:** Estimates of Sensitivity/Specificity of VP7 Blocking ELISA

Sensitivity estimate									
Animal status	ExV	*n*	Pos	Neg	Average sensitivity	95% CI LL	95% CI UL	Min	Max
Vaccinated and not infected with AHSV									
Vaccinated animals with attenuated vaccine	POS	336	317	19	94.3%	92.9%	97.7%	88.1%	100.0%
Vaccinated animals with inactivated vaccine	POS	248	236	12	95.2%	91.7%	97.5%	90.3%	100.0%
Vaccinated animals with sub‐unit/MVA‐VP2* vaccine	POS	8	8	0	100.0%	63.1%	100.0%	100.0%	100.0%
Overall sensitivity	POS	592	561	31	94.8%	93.5%	97.1%	89.2%	100.0%
Infected with AHSV									
Naturally or experimentally infected with AHS	POS	184	181	3	98.4%	95.3%	99.7%	95.7%	100.0%
Vaccinated and experimentally infected with AHS									
Vaccinated with attenuated vaccine and experimentally infected	POS	88	88	0	100.0%	95.9%	100.0%	100.0%	100.0%
Vaccinated with inactivated vaccine and experimentally infected	POS	136	136	0	100.0%	97.3%	100.0%	100.0%	100.0%
Vaccinated with MVA‐VP2* vaccine and experimentally infected	POS	32	11	21	34.4%	18.6%	53.2%	25.0%	50.0%
Vaccinated with all vaccine types and experimentally infected	POS	256	235	21	91.8%	85.9%	93.5%	90.6%	93.8%
Overall sensitivity on infected animals	POS	440	416	24	94.5%	90.8%	95.6%	92.7%	96.4%
Overall sensitivity estimate	POS	1032	977	55	94.7%	93.1%	96.0%	90.7%	98.4%

Specificity estimate									
Animal status	ExV	*n*	Pos	Neg	Average specificity	95% CI LL	95% CI UL	Min	Max
Naïve animals (AHSV noninfected)	NEG	328	0	328	100.0%	98.9%	100.0%	100.0%	100.0%
Vaccinated and not infected with AHSV	NEG	128	4	124	96.9%	92.2%	99.1%	87.5%	100.0%
Overall specificity estimate	NEG	456	4	452	99.1%	97.8%	99.8%	96.5%	100.0%

Note

95% CI LL: confidence interval 95% low limit; 95% CI UL: confidence interval 95% low limit; ExV: expected value according to previous ELISA results; Max: maximum value; Min: minimum value; MVA: Modified Vaccinia Ankara viruses expressing single African horse sickness virus VP2 antigens; *n*: number of observations.

## 4 DISCUSSION

The clinical outcome of AHS in horses is characterized by a rapid evolution of the disease inducing syndromes that range from subacute to per‐acute form. Mortality rates typically exceed 50%, but can reach 90% depending on clinical form of the disease and the immunological status of the animals (Mellor & Hamblin, 2004). The infected horse often dies shortly after infection, and thus there is limited opportunity to mount a strong antibody response. The use of attenuated vaccines in endemic areas helps prevent horses from succumbing to AHS, but makes it difficult to determine whether AHS‐specific antibodies are derived from an immune response to the vaccine or to the AHSV causing the outbreak. This situation is even more complicated in endemic countries where polyvalent vaccines are used and different AHSV serotypes circulate in the field. The difficulty of gathering a number of well characterized sera to validate the serological tests and assess their fitness for the different purposes for which the tests are to be used is an important consideration for the preparation of the serum panel used in our study. A significant number of sera representing the various epidemiological situations of the disease were collected. This, together with the organization of an international collaborative trial among reference and specialist AHS laboratories, provided the opportunity to obtain robust and reliable data on the test's reproducibility (Stage 3 of the Validation Pathway). It is important to note that for our study we used a commercial diagnostic ELISA with well characterized diagnostic performance characteristics, that had already been assessed according to the Stage 1 and Stage 2 of the OIE validation pathway (OIE, 2012).

Expression of AHS‐VP7 proteins by means of recombinant baculoviruses has been used to manufacture large quantities of immunodiagnostic reagents of good quality. Previous studies have used this recombinant antigen in indirect ELISA procedures, demonstrating that VP7‐based antibody detection methods are rapid, robust and highly accurate diagnostic tools, with superior diagnostic accuracy to the classical virus neutralization test (Maree & Paweska, 2005).

ELISA methods based on the competitive‐blocking principle are useful for the diagnosis of viral and bacterial diseases (Gallardo et al., 2015; Praud et al., 2016) due to their good diagnostic performance and their potential to be used with sera independent of the animal species of origin. Currently, the VP7 Blocking ELISA for the diagnosis of AHS is available as a commercial kit at National Reference Laboratories of the EU, and its performance is checked annually through ring trials organized by the European Reference Laboratory for AHS (Agüero, personal communications). In many laboratories, the test is currently the basis of preexport testing for AHS, a procedure that is routinely used to support the intense and increasing movement of horses across international borders. Both indirect and blocking VP7 ELISA methods are tests recommended by OIE for serological diagnosis of AHS (OIE, 2017b) and are prescribed tests by European legislation for movement checks and importation of *Equidae* (European Union, 2009).

We conducted a thorough analysis of performance of the AHS‐VP7 Blocking ELISA with the view of generating enough data to support the validation of this assay up to Stage 3 (Reproducibility) of the OIE validation pathway for diagnostic tests (OIE, 2012, 2017c,d). Data from one laboratory clearly performed as an outlier and, after investigation, was excluded from the assessment due to unidentified operational errors, after which, reproducibility was estimated by assessing the overall sensitivity and specificity and their associated uncertainty.

The good test sensitivity (98.4% [95% CI: 95.3–99.7]) of the VP7 Blocking ELISA in naturally or experimentally infected animals confirms its ability to detect nonvaccinated individuals previously exposed to virus and would support its use for trade purposes. The ability to detect antibodies in infected horses previously immunized with vaccines containing complete AHSV virions was also very good either in animals vaccinated with attenuated (100% [95% CI: 95.9–100]) or with inactivated vaccines (100% [95% CI: 97.3–100]). This reveals the potential usefulness of the test to assess the sero‐prevalence of AHS in a population of *Equidae*. In the case of a population where vaccination was performed, with either inactivated or live attenuated vaccines, it would be difficult to determine whether the seropositive animals had been exposed to field virus. In these cases, further investigations would be required, which could potentially include the routine monitoring of the serological status of the population (or of sentinel herds) or examination of the vaccination and clinical histories of the animals. The utility of serological methods for these purposes needs further analysis and evaluation, and the good performance of the VP7 Blocking ELISA would be a good candidate to determine the value of serology as a surveillance tool in endemic countries. In the case of a nonendemic country, the high sensitivity and specificity of the test indicate that the VP7 ELISA is an excellent tool for disease surveillance and corroboration of disease freedom once infection occurring during an outbreak has been eradicated.

In our study we computed any dubious result as positive. Of the total of results computed in the calculations of sensitivity and specificity (*n* = 1032), only 47 (4.6%) were classified as dubious. This very low percentage of dubious results is unlikely to affect negatively the use of this assay for surveillance procedures. In practice, it would be appropriate to initially classify any dubious results as provisional positives that require further investigation depending on specific epizootiological circumstances (epizootic in nonendemic country, epizootic in endemic country, posteradication surveillance in nonendemic country etc.…)

Several initiatives to develop DIVA (Differentiation of Infected from Vaccinated Animals) vaccines have been developed over the years due to the unsuitability of current vaccines to discriminate between active AHS‐infected and ‐vaccinated horses in a population. Some potential DIVA vaccines are based on the immunogenic antigen VP2 or VP2 and VP5 (Alberca et al., 2014; Castillo‐Olivares et al., 2011; Chiam et al., 2009; Guthrie et al., 2009), which are the targets of virus neutralizing antibodies of the host. Because such vaccines lack VP7 in their composition, they would not induce a VP7 antibody response and therefore would not interfere with current diagnostic tests based on VP7. A combination of these VP2/VP5‐ based vaccines with VP7 diagnostic tests would offer useful DIVA capacity and would improve surveillance procedures. With this in mind we included in the panel some serum samples from horses vaccinated and experimentally infected with AHS. Thus, we also assessed the VP7 Blocking ELISA performance in animals experimentally infected with AHSV after being vaccinated with MVA viruses expressing single AHSV VP2 antigens (Alberca et al., 2014). Although the animals were fully protected against the disease, the level of VP7 antibodies detected was low. This finding would be consistent with immune response of individuals with high levels of protection against AHSV in which exposure to the virus (and VP7 antigen) is significantly reduced in terms of time and intensity. The availability of this type of sample was very limited, but the results of our study demonstrated the potential of VP7 Blocking ELISA to be used in combination with VP2‐based DIVA vaccines. Further work is needed to corroborate the applicability of the VP7 Blocking ELISA as a DIVA test when it is used with VP2‐based vaccines.

The test also showed good capacity to monitor the response to vaccination because of its capacity to detect antibodies in vaccinated noninfected horses (94.8%; 95% CI: 93.5–97.1), with no major differences between types of vaccine used. This is extremely important when organizing preventive sanitary campaigns in order to monitor the operational activities of field vaccination programmes. The values obtained in our study are in line with those obtained by previous work when evaluating the VP7‐based indirect ELISA in its ability to detect horses immunized with attenuated vaccine (Maree & Paweska, 2005). In our study, a number of serum samples that were derived from vaccinated and not infected horses produced a negative result by the VP7 Blocking ELISA (this results coincided with the original results obtained at the laboratory of origin). This can be explained from the fact that these sera were collected during the last years of the eradication campaign of the last AHS Spanish outbreak (1987–1992), when AHSV was not circulating in the field, long time after the animals were last vaccinated (as indicated in Table 1). Therefore, under these circumstances, it is not surprising that the antibody titres had waned to undetectable levels by the time the horses were sampled.

The surveillance of AHSV free territories is of great importance in containing the disease within endemic areas. For this reason, serological diagnostic tests need to be highly sensitive to accurately detect possible incursions of AHSV in nonendemic areas or to certify with confidence that a previously infected population is free of AHS after the implementation of an eradication programme. In addition, they need to be highly specific to avoid the unnecessary costs associated with the animal movement restrictions that would be imposed in the region until investigation of any false positive results is concluded. The results on test specificity obtained with VP7 Blocking ELISA in our study (100% [95% CI: 98.9–100]) combined with its high sensitivity (reviewed above) would suggest its feasibility for the surveillance of the AHS in free areas. Again, the values obtained in our study are in line with those obtained by previous work when evaluating the VP7‐based indirect ELISA (Maree & Paweska, 2005).

In conclusion, through this comprehensive evaluation we can conclude that the VP7 Blocking ELISA is reproducible and satisfies the OIE requirements of reproducibility. However, it is recommended (OIE, 2012, 2017c) that monitoring of diagnostic performance of the assay is periodically performed through international ring trials, such as those that have been previously conducted by the OIE/EU reference laboratories over the years.

The VP7 Blocking ELISA, in its commercial version, is ready to enter Stage 4 of the validation pathway (Programme Implementation). Specifically, future studies should aim to test the diagnostic performance of the assay using contemporary serum samples collected during control campaigns in endemic countries. This type of analysis will help to determine positive and negative predictive values of the tests in the various epidemiological scenarios in endemic territories.

## CONFLICT OF INTEREST

P. Rueda and P. Sastre are employees of INGENASA, the manufacturing company of the AHS Blocking ELISA test, but this fact does not represent in itself a competing interest. INGENASA participated by supplying the test kits, and performing the ELISA tests on the samples from the panel, which were blindly coded. Interpretation and analysis of the data, collected from nine different laboratories, including INGENASA, was conducted by Drs Duran, Aguero, Gubbins and Castillo‐Olivares. We therefore conclude that the objectivity of the study was not affected. Otherwise there are no competing interests.
